# Extracurricular activity participation moderates impact of family and school factors on adolescents’ disruptive behavioural problems

**DOI:** 10.1186/s12889-015-2464-0

**Published:** 2015-11-11

**Authors:** Corine M.E.F. Driessens

**Affiliations:** Department of Social Sciences, University of Southampton, Highfield campus, Southampton, UK

**Keywords:** Internalizing behaviour, Externalizing behaviour, Extracurricular activities, Longitudinal research, Generalized estimating equation

## Abstract

**Background:**

The prevalence of problem behaviours among British adolescents has increased in the past decades. Following Erikson’s psychosocial developmental theory and Bronfenbrenner’s developmental ecological model, it was hypothesized that youth problem behaviour is shaped in part by social environment. The aim of this project was to explore potential protective factors within the social environment of British youth’s for the presentation of disruptive behavioural problems.

**Method:**

This study used secondary data from the Longitudinal Study of Young People in England, a cohort study of secondary school students. These data were analysed with generalized estimation equations to take the correlation between the longitudinal observations into account. Three models were built. The first model determined the effect of family, school, and extracurricular setting on presentation of disruptive behavioural problems. The second model expanded the first model by assuming extracurricular activities as protective factors that moderated the interaction between family and school factors with disruptive behavioural problems. The third model described the effect of prior disruptive behaviour on current disruptive behaviour.

**Results:**

Associations were found between school factors, family factors, involvement in extracurricular activities and presence of disruptive behavioural problems. Results from the second generalized estimating equation (GEE) logistic regression models indicated that extracurricular activities buffered the impact of school and family factors on the presence of disruptive behavioural problems. For instance, participation in sports activities decreased the effect of bullying on psychological distress. Results from the third model indicated that prior acts of disruptive behaviour reinforced current disruptive behaviour.

**Conclusion:**

This study supports Erikson’s psychosocial developmental theory and Bronfenbrenner’s developmental ecological model; social environment did influence the presence of disruptive behavioural problems for British adolescents. The potential of extracurricular activities to intervention strategies addressing disruptive behavioural problems of adolescents is discussed.

## Background

Disruptive behavioural problems have become more common among British adolescents over the past 30 years [1]. In 2004, about 5 % of girls and 9 % of boys reported an externalizing behaviour problem such as conduct disorder [2] while, in 2006, about 20 % of teenage girls and 7 % of teenage boys reported an internalizing behaviour problem such as anxiety and/or depression [3]. About 20–30 % of young people with behavioural problems had tried to harm themselves or commit suicide [2]. Among adolescents included in the British 1946 birth cohort, externalizing behavioural problems during adolescence (age 13–15) were shown to affect development into adulthood as they were associated with increased teenage parenthood (before age 20), increased divorce rate (age 35, 43, and 53), decreased school qualifications (age 26), and increased adult alcohol abuse (age 43 and 53) [4].

The literature suggests that social environmental factors help shape adolescent problem behaviour. For instance, research has shown that disruptive behavioural problems are associated with parental separation, parental mental illness, and loss of important friendships [5, 6]. Following Erikson’s psychosocial developmental theory [7], it has been argued that social interactions within household, school, and community provide the experiences, information, encouragement, and reinforcement the adolescent will use to develop a sense of self and feelings of independence and control. Through their activities adolescents develop their interests, discover their talents, and become committed to certain values and beliefs [8–10]. Whether social interactions facilitate, maintain, or impede positive youth development depends on the type and frequency of the social activity as well as the quality of the social interchange [11]. Social interchanges occur in a variety of settings [12]. This paper is focussing on adolescent’s social activities within extracurricular activity, family, and school settings.

### Social activities in extracurricular activity setting

Previous studies have shown that young people with positive ties to extracurricular activities were less likely to be involved in substance use [13, 14] or to display other disruptive behaviours [15, 16]. These adolescents reported higher levels of well-being [17]. For instance, participation in religious activity was associated with emotional regulation [18]. Volunteer activities were not only related to lower levels of internalizing/externalizing behaviour [19], but also to lowered teenage pregnancy and suspension rates [20].

Whether and to what extent participation in extracurricular activity is beneficial may depend on the specific activity and disruptive behaviour considered. For example, team sports participation has been linked positively with alcohol consumption [15] and smoking [21], but negatively with depression and suicide [8, 22]. Similarly, volunteering has been negatively associated with externalizing disruptive behaviours [23], but has not shown an association with internalizing disruptive behaviours [24]. In addition, a cross-sectional study found a positive relationship between participation in art activities and alcohol/drug use [8], but looking at art involvement over time an association was found with lower substance use [25].

Not all studies find a relationship between participation in extracurricular activities and internalizing/externalizing behaviour [26, 27]. These discrepancies may be explained by differences in conceptualization of extracurricular activities [28].

### Social activities in family setting

Family structure and relationships are important factors related to adolescent disruptive problem behaviour. Family structure research has focussed on the presence of biological parents and the number of siblings [29]. Living in an intact family lowered the risk for onset of problem behaviour [30], while adolescents living apart from their biological father were more likely to engage in disruptive problem behaviours [31].

Family relationship research has shown that parental supervision, guidance, and connectedness facilitated adolescent social development, promoted a sense of well-being, and decreased the risk for internalizing and externalizing behavioural problems [30, 32, 33]. Quality of the parent-adolescent relationship and parental involvement in the adolescent’s schooling have been identified as predictors of adolescent mental health [34, 35]. Supportive and close relationships reduced the risk of problem behaviour. However, hostility and conflict within the parent-adolescent relationship increased the risk of presenting with internalizing and externalizing problem behaviours [29, 36].

### Social activities in school setting

Interaction between peers have been identified as a very important social indicator of adolescent well-being [37–39]. For instance, extensive research has shown that bullying within the school setting has serious long-term consequences, in the form of lifetime internalizing/externalizing behaviours, repeated suspension from school and work, and heightened suicide risk [40, 41]. Personal characteristics associated with bully victims include lack of social skills, negative self-related cognitions, and poor social problem solving skills [42].

### Overview of the present study

Based on the above-described studies extracurricular activities appear to have the potential to work protectively against the development of disruptive behavioural problems during adolescence. The presence and strength of the relationship between extracurricular activity and youth developmental outcome, however, depend on the activity and the outcome being studied. Potential protective social interchanges within the adolescent’s family and school settings have also been identified. Against this background, the current study expanded prior research by employing Bronfenbrenner’s developmental-ecological model. This model [43] states that adolescents’ social interchanges are not only affected by the setting in which they occur but also by their social experiences in other settings [44]. This model hypothesizes an interaction between social interchanges in different settings. The interaction investigated in the current study determined whether involvement in extracurricular activities could moderate the effect of family and school social interchanges on disruptive behavioural problems during secondary school. Family interchanges considered in this study covered family structure and parent-adolescent relationship. School interchanges were limited to bullying. Any constructive use of leisure time was considered an extracurricular activity. For example, the definition for volunteering used in this study, as coined by Wilson [23], recognized formal and informal proactive helping behaviour in which time was given freely to benefit another person, group, or organization.

## Methods

### Participants & procedure

The Longitudinal Study of Young People in England (LSYPE) is a prospective study following a representative sample of young people attending year nine (13/14 year olds) in England [45]. Data collection started in 2004 and occurred on a yearly basis thereafter until 2010. The secondary dataset made publicly available contains adolescents’ interviews covering 2004 to 2010 and parental interviews covering 2004 to 2007. The data are unique in having repeated measures of disruptive behavioural problems in a representative sample of English adolescents. While the LSYPE has a wealth of measures suitable for defining disruptive behavioural problems, extracurricular activities, family factors, and school factors, only a few of these measures are consistently available over time. Therefore, the data used came from the first four waves of the LSYPE. The response rate of parents and/or young persons who completed their full interview was respectively 66 % (13695 students age 13/14), 76 % (11,965 students age 14/15), 90 % (12,168 students age 15/16), and 89 % (11076 adolescents age 16/17). The sample in wave 4 included an ethnic boost of 309 ethnic adolescents, which was not included in this study.

The LSYPE sample included about 10 per cent of adolescents diagnosed with a disability, long-standing illness or suffering from a long-standing infirmity. For about half of these adolescents this condition limited their activities. Initial modelling showed that this disability status was related with participation in extracurricular activity, school and family factors, as well as disruptive behavioural problems. This interaction effect of disability status was outside the scope of this study. Therefore, those adolescents with long-standing illness/infirmity or disability were excluded from the analyses.

## Measures

### Disruptive behaviour

#### Internalizing behaviour

Internalizing behaviour was operationalized using the 12-item scale of the Generalized Health Questionnaire (GHQ-12). This scale measured psychiatric morbidity among the adolescents of the LSYPE study [46]. This scale included six questions that were positively worded (e.g. felt you were playing a useful part in things, felt capable of making decisions, able to enjoy your day-to-day activities). The response scale for the positively worded questions ran from ‘more so than usual’ (1) to ‘much less than usual’ (4). Six items in this scale were negatively worded (e.g. felt constantly under strain, losing confidence in yourself, been thinking of yourself as a worthless person). The response scale for the negatively worded questions ran from ‘not at all’ (1) to ‘much more than usual’ (4). Higher scores on the GHQ-12 indicated greater levels of general psychiatric distress. This scale was developed as a screening instrument and its performance has been validated on its sensitivity and specificity to identify people experiencing distressing symptoms who might benefit from psychological or psychiatric treatment [46]. Among adolescents a cut-off of 11 indicated psychological distress and a cut-off of 20 indicated psychiatric distress [47]. The reliability of this measure in the current study (Cronbach’s alpha = .86) was consistent with reports from other studies (.82–.87).

#### Externalizing behaviour

The following four indicators of externalizing behaviour were assessed in the LSYPE [48]: (1) Substance use measures (“ever try cannabis?” (yes/no) as well as frequency alcohol consumption and cigarette use) were chosen to correspond to items used in a major cross-sectional UK government study on substance use [49]. More than six cigarettes per week and alcohol consumption on most days were coded as “problem behaviour”. There is medium reliability (Kuder-Richardson formula 20 of 0.50) between the three items. Between 3 and 11 % of all participants used more than one substance at a time. (2) Absenteeism was measured using reports on suspension, expulsion, and truancy (yes/no). Parental and young person’s report were assessed at the first three waves. Due to this mix of source information, the items measured during wave 1, wave 2, and wave 3 only had low reliability (Kuder-Richardson formula 20 ranges between 0.34 and 0.44). Only about 5–7 % of the participants experienced more than one form of absenteeism. (3) Delinquency was measured with four items: spray painting on walls, breaking public property, shoplifting, and public fighting (yes/no). A composite score of these items resulted in a fair reliability (0.65). (4) Parental perception of the young person’s interaction with authority was measured by reports on interaction with police, local council social service, educational welfare service, or other similar type of service (yes/no). A composite score of these items resulted in a reliability ranging between 0.66 and 0.68.

### Extracurricular activities

The extracurricular activities included in this study were school-based and community based after-school activities. The young person’s interview assessed participation in these activities with three items. First, participation in sports, music, religious, youth club, and formal volunteering activities was assessed using the question: “Here is a list of things people can do when they are not in school, can you tell me which, if any, you have been doing in the past four weeks?”. Regretfully, the youth’s report on participation in sports, music, religious, and youth club activities was not assessed for wave 3. Second, the frequency of participation in sports and religious activities was recorded (never, less than once a month on average, once or twice a week, 3–4 times a week, 5+ a week). Third, informal volunteering was assessed using the question “some people your age may have to look after other people. This could be a brother or sister, children under 14 who live outside your household, an elderly person, or someone who is disabled or sick. Is there anyone like this that you have to look after on a regular basis without pay?”.

Young person reports were supplemented with parental reports. Parental reports included questions on expressive, sports, and religious community-based organized activities and frequency of attendance. The extracurricular activities assessed by parental and young person’s interview were used to represent sports, (formal and informal) volunteering, expressive (music, dance, drama), religious, and youth club extracurricular activities. Differences in measurement scales of the extracurricular activities was corrected by coding participation on a regular basis (e.g. once a month or more) as ‘yes’ and infrequent or no participation as ‘no’.

### Family factors

The family structure was measured with indicators including parental composition (couple, versus single/no parents) and family size (0, 1–2, and 3+ siblings).

#### Parental involvement in education

Parental involvement in education was measured with two different items: “how often they speak with teachers” (once a week, once every 2–3 weeks, once a term, less than once a term, never) and “how involved they feel in the adolescent’s education” (very involved, fairly involved, not very involved, never involved). The rank correlation (Goodman and Kruskal gamma) between these items ranged between .24 and .28. Taking the difference in measurement scale into account, an average score for parental involvement was used. Parental involvement was quite stable over the first three waves, with 76 % of the parents not changing their involvement level. A variable representing average parental involvement in past was created to substitute missing data in wave 4.

#### Parent-adolescent relationship

This construct was assessed using two different items: “frequency of parent–child arguments” (most days, more than once a week, less than once a week, hardly ever, never) and “quality of parent–child relationship” (very well, fairly well, fairly bad, bad). The rank correlation (Goodman and Kruskal gamma) between these items ranged between .54 and .58. Taking the difference of measurement scale into account, a dichotomized variable was created from the average score on these two items; “good relationship (well/fairly well relationship) and “bad relationships” (fairly bad/bad relationship). Parent–child conflict data was missing in wave 4. The respondents’ mode for prior parent–child relationship was used to substitute this missing data.

### School factors

#### Bullying

Bullying was assessed with four yes/no questions identified by Mynard, & Joseph [50] as the main types of bullying (being called hurtful names, been forced to give other students money, threatened with violence, been a victim of violence). The reliability of this measure was acceptable (.58–.67). A variable was created indicating the number of different types of bullying the respondent was exposed to (none – four types of bullying).

### Statistical modelling

The hypothesis under investigation focussed on the association between social interchanges in different settings and presence of disruptive behavioural problems for the adolescent population of England. This hypothesis was extended by studying the moderating effect of participation in extracurricular activities on the association of social interchanges in family and school setting and presence of disruptive behavioural problems for the adolescent population in England. Population average coefficients were obtained by modelling the longitudinal data. Generalized Estimation Equations (GEE) were chosen to estimate the average population trend while accounting for longitudinal dependence [51]. In simulation studies comparing different types of missing data (Missing At Random [MAR] versus Missing Completely At Random [MCAR]), GEE models showed near identical results, thereby providing justification for usage where data is MAR [52].

Due to the design of the LSYPE there were many different types of non-response (school non-participation, students and/or parents non-participation, or item non-response). Factors associated with non-response at baseline consisted of ethnicity, gender, region, school deprivation status, and GCSE performance. Non-response at follow-up was associated with ethnicity, school deprivation status, computer in household, continued attendance to school, parental government benefit claim status, and parental education. Generalizability of longitudinal analyses was affected by these non-response-associated selectivity effects. To compensate for non-response bias, a cross-sectional wave 1 weight was used to adjust the data to a representative sample of adolescents in 2004 [53]. To check if this weight adjustment was the appropriate way to reduce bias, results were compared with models that used no weight or longitudinal wave 1 – wave 4 weight. Use of cross-sectional and longitudinal weight gave comparable results. Ethnic differences were more pronounced in the unweighted models as initial sampling procedures of LSYPE included oversampling of minority groups. This verification indicated that results with cross-sectional weight adjustment were stable.

In addition to weight adjustment of the statistical models, longitudinal non-response was also addressed with the use of a GEE approach to modelling. By clustering the individual’s responses at multiple time points each response was treated as a separate observation, but adjustments were made for correlation between the observations. Clustering also allowed incomplete data to be included. Thus, not only participants who participated in all four waves were included in the analyses, but the response of the young people who participated once, twice, or three times was also considered. Although this study accounted for the individual-level cluster correlation, school-level cluster correlations could not be accounted for as this type of information was not publicly available in the LSYPE dataset.

Maximum likelihood estimates were obtained by an algorithm known as iterative generalized least squares. The structure of the correlation matrix specified is known as the Toeplitz matrix [54]. The diagonal elements of this matrix were a function of the predicted probability from the previous iteration. The off-diagonal elements were a function of the correlation among the observations. One correlation was used for measurements that were one time-point apart. Another correlation was used for measurements that were two time-points apart. Again another correlation was used for the measurements that were three time-points apart

Initial modelling explored the potential association of each individual predictor with each disruptive behavioural problem. Subsequently, only significant extracurricular, family, and school predictors were included in the logistic regression models. Model 1 was a full model entering all significant potential predictors (extracurricular activities, family factors, and school factors) into the same model. Model 2 tested for interaction effects between significant extracurricular activities and significant family or significant school factors. Model 3 described the relationship among the repeated outcome measures (alternating logistic regression) [55]. This model employed the exchangeable model of the logarithm of odds ratio. This means it calculated a single odds ratio for all pairs of repeated measurements controlling for the significant predictors of disruptive behavioural problems. All models controlled for individual characteristics (sex, ethnicity, religion) and family economic resources such as housing (owner versus rented), social class (manual versus higher social class), household yearly income (< £12480, £12480–£31200, > £31200), and parental qualifications (higher education degree, General Certificate of Education {GCE}, General Certificate for Secondary Education {GCSE}, and qualification for basic knowledge and skill training & no qualification).

## Results

An overview of the descriptive statistics is presented in Table 1. Three times as many adolescents reporting substance use in 2007 compared to 2004. The descriptive statistics in Table 1 suggest a change in extracurricular interest with increasing age. Fewer adolescents reported participation in youth clubs and expressive activities over time while more adolescents reported involvement in volunteering. Family structure and economic resources were quite stable over time, but changes were visible for parental involvement in school over time and the number of adolescents reporting bad parent–child relationship over time.

**Table 1:** Descriptive statistics and collection methods used for the study variables at each wave

Study variables	% @ T_1_	% @ T_2_	% @ T_3_	% @ T_4_
	N^a^ = 11 868	N^a^ = 12 111	N^a^ = 13 914	N^a^ = 13 085
*Outcome*				
Mental well-being				
Psychological distress		32.4		33.1
Psychiatric distress		6.7		6.9
Substance use	12.9	25.2	32.0	39.9
Interaction authority	13.3	12.6	11.8	
Delinquency	29.0	24.3	21.1	
Absenteeism	40.0	46.0	39.6	
*Personal characteristic*				
Sex (female)	50.3	50.1	49.2	49.1
Ethnicity				
White	87.7	87.7	88.4	88.2
Indian	5.2	5.2	4.8	5.0
Mixed	2.9	3.0	2.9	2.9
Other	4.2	4.1	3.8	3.9
Religion				
None	37.5	37.7	37.7	37.8
Christian	52.5	52.4	53.1	52.8
Muslim	6.2	6.2	5.7	5.9
Other	3.8	3.8	3.5	3.5
Parental qualifications				
Degree	18.6	18.5	18.1	18.0
GCE	33.7	33.5	33.7	33.7
GCSE	26.3	26.4	26.4	26.4
Level 1 or below	21.5	21.7	21.8	21.8
Social class				
High occupation/Professional	41.4	41.9	43.8	41.0
Intermediate occupation	7.3	7.0	7.2	9.4
Small occupation/self-employed	13.0	9.1	7.0	11.4
Supervisory & technical	12.0	13.1	13.2	9.8
(Semi-) routine occupation	21.2	24.5	25.5	25.9
Not employed	5.1	4.4	3.3	2.5
Household income				
Below poverty	27.1	27.3	27.4	27.4
Middle	41.3	41.4	41.6	41.5
Highest quartile	31.6	31.4	31.0	31.1
Housing (renting)	26.8	24.5	24.4	22.5
*Extracurricular activity*				
Volunteering	8.1	10.8	5.9	16.8
Religious activities	17.9	15.4	10.2	15.6
Expressive activities	29.5	25.8	13.7	21.5
Youth club activities	20.7	17.0	11.4	14.9
Sports activities	88.6	85.4	93.7	72.1
*Family factors*				
Parental composition				
Single/no parent	26.2	23.6	24.9	29.0
Number of siblings				
0	13.5	14.4	16.3	15.9
1–2	66.3	66.3	65.8	65.7
3+	20.2	19.2	17.9	18.4
Parent-adolescent relationship (bad)	6.3	12.6	16.8	11.1
*School factors*				
Bully victim				
No	62.7	68.7	78.2	67.1
1 type	19.8	16.5	12.1	15.4
2 types	9.7	8.9	6.3	10.5
3 or 4 types	7.8	6	3.4	7.1

T_1_-T_4_ represents 2004 – 2007. ^a^number of participants adjusted with cross-sectional weight to minimize bias

### Model 1

The hypothesis tested in this model was that extracurricular, family, and school factors could be classified as protective factors for disruptive behavioural problems. Only variables significant in initial modelling were entered into model 1 (Tables 2 and 3). A negative coefficient indicated a greater likelihood (odds between zero and one) of no disruptive behavioural problems. A positive coefficient indicated a greater likelihood (odds > 1) of the presence of disruptive behavioural problems.

**Table 2:** Impact of baseline internalizing behavioural problems, extracurricular activities, family, and school factors on internalizing behavioural problems

		Psychological distress				Psychiatric distress			
	Measures	*β*	SE	OR	95 % CI	*β*	SE	OR	95 % CI
Model 1	*Extracurricular activity*								
	Sports (yes)	-.16^**^	.05	.85	.77–.94	-.25^*^	.10	.78	.64–.94
	Volunteering (yes)	-.02	.06	.98	.88–1.1				
	Expressive (yes)	.25^***^	.05	1.3	1.2–1.4	.25^*^	.10	1.3	1.1–1.6
	Religious club (yes)					.31^**^	.11	1.4	1.1–1.7
	Youth club (yes)	-.11	.06	.90	.80–1.0				
	*Family factors*								
	Parent Status (single)	.11^*^	.05	1.1	1.0–1.2	.01	.10	1.0	.82–1.2
	Parent-adolescent relationship (bad)	.36^***^	.07	1.4	1.2–1.6	.45^***^	.12	1.6	1.3–2.0
	*School factors*								
	Bully victim (no)								
	1 type	.76^***^	.05	2.1	1.9–2.4	1.1^***^	.11	3.0	2.4–3.7
	2 types	.88^***^	.06	2.4	2.1–2.7	1.3^***^	.12	3.7	2.9–4.7
	3 or 4 types	1.4^***^	.07	4.0	3.5–4.7	1.9^***^	.12	6.9	5.4–8.8
	**Moderating effect**								
Model 2	*Sport*								
	Parent-adolescent relationship								
	Good	-.21^***^	.05	.81	.73–.90	-.27^*^	.11	.76	.62–.94
	Bad	.17	.13	1.2	.92–1.5	.18	.24	.84	.52–1.3
	Bully Victim (no)	-.09	.06	.91	.81–1.0	-.21	.14	.81	.61–1.1
	1 type	-.28^*^	.11	.76	.61–.94	-.33	.19	.72	.50–1.0
	2 types	-.43^**^	.14	.65	.50–.86	-.01	.22	1.0	.65–1.5
	3 or 4 types	-.07	.16	.93	.68–1.3	-.46^*^	.22	.63	.41–.97
	*Expressive*								
	Parent-adolescent relationship								
	Good	.22^***^	.05	1.3	1.2–1.4	.19	.11	1.2	.91–1.5
	Bad	.48^**^	.15	1.6	1.2–2.2	.65^**^	.25	1.9	1.2–3.1
	Bully Victim (no)	.26^***^	.07	1.3	1.3–1.5	.28	.16	1.3	.98–1.8
	1 type	.29^**^	.10	1.3	1.1–1.6	.25	.18	1.3	.89–1.8
	2 types	.13	.12	1.1	.95–1.4	.33	.22	1.4	.91–2.1
	3 or 4 types	.24	.14	1.3	.96–1.7	.12	.22	1.1	.74–1.7
	*Religious*								
	Parent-adolescent relationship								
	Good					.34^**^	.12	1.4	1.1–1.8
	Bad					.16	.28	1.2	.74–2.0
	Bully Victim (no)					.45^**^	.16	1.6	1.1–2.1
	1 type					.29	.22	1.3	.86–2.1
	2 types					.28	.28	1.3	.76–2.3
	3 or 4 types					.10	.24	1.1	.69–1.8
	**Baseline behaviour**								
Model 3	Psychological distress	1.2^***^	.07	3.2	2.8–3.7				
	Psychiatric distress					1.4^***^	.20	4.1	2.8–6.2

Models are controlling for sex, ethnicity, religion, parental education, household income, housing, and social class; significance is shown in the table ^*^ *p* < .05 ^**^ *p* < .01 ^***^ *p* < .001

**Table 3:** Impact of baseline externalizing behavioural problems, extracurricular activities, family, and school factors on externalizing behavioural problems

		Substance use				Authority				Delinquent				Absenteeism			
	Measures	*β*	SE	OR	95 % CI	*β*	SE	OR	95 % CI	*β*	SE	OR	95 % CI	*β*	SE	OR	95 % CI
Model 1	*Extracurricular Activity*																
	Sport (yes)	-.13^*^	.05	.87	.78–.97	-.34^**^	.12	.71	.56–.91					.21^**^	.07	1.2	1.1–.1.4
	Expressive (yes)					-.41^***^	.11	.66	.54–.82	-.23^***^	.06	.80	.70–.90	-.23^***^	.06	.79	.70–.88
	Religious club (yes)	-.21^***^	.06	.81	.72–.92	-.25^*^	.12	.78	.61–.99	-.18^*^	.08	.83	.71–.98	-.15^*^	.07	.86	.75–.99
	Youth club (yes)					-.02	.10	.98	.80–1.2	.09	.07	1.1	.96–1.2				
	*Family factors*																
	Parent Status (single)	.37^***^	.06	1.4	1.3–1.6	.40^***^	.11	1.5	1.2–1.8	.16^*^	.08	1.2	1.0–1.4	.21^**^	.07	1.2	1.1–1.4
	Siblings – none																
	1/2 siblings					-.01	.13	.99	.77–1.3	.02	.09	1.0	.86–1.2	-.03	.08	.97	.84–1.1
	3+ siblings					.25	.16	1.3	.94–1.7	.17	.11	1.2	.95–1.5	.23^*^	.10	1.3	1.0–1.5
	Parent-adolescent relationship (bad)	.54^***^	.07	1.7	1.5–2.0	1.0^***^	.13	2.7	2.1–3.5	.56^***^	.10	1.7	1.4–2.1	.52^***^	.09	1.7	1.4–2.0
	Level involvement	.12^*^	.04	1.1	1.1–1.2	.48^***^	.07	1.6	1.4–1.8	.15^***^	.05	1.2	1.1–1.3	.09^*^	.04	1.1	1.0–1.2
	*School factors*																
	Bully Victim – no																
	1 type	.48^***^	.05	1.6	1.5–1.8	.26^*^	.10	1.3	1.1–1.6	.41^***^	.07	1.5	1.3–1.7	.23^***^	.06	1.3	1.1–1.4
	2 types	.63^***^	.06	1.9	1.7–2.1	.45^***^	.13	1.6	1.2–2.0	.79^***^	.08	2.2	1.9–2.6	.59^***^	.08	1.8	1.5–2.1
	3 or 4 types	.86^***^	.07	2.4	2.0–2.7	.85^***^	.13	2.3	1.8–3.0	.85^***^	.10	2.4	2.0–2.9	.58^***^	.09	1.8	1.5–2.1
	**Moderating effect**																
Model 2	*Sport*																
	Parent-adolescent relationship																
	Good	-.11	.06	.89	.79–1.0	-.43^***^	.13	.65	.51–.83					-.21^**^	.08	.81	.70–.94
	Bad	-.26	.14	.77	.59–1.0	.38	.37	1.5	.71–3.0					-.21	.26	.81	.49–1.4
	Parent status																
	Single	-.06	.09	.95	.79–1.1	-.40	.21	.67	.44–1.0					-.18	.14	.84	.63–1.1
	Couple	-.17^**^	.07	.84	.74–.96	-.31^*^	.15	.74	.55–.99					-.22^**^	.08	.80	.68–.95
	Siblings																
	0					-.96^***^	.28	.38	.22–.66					-.31	.16	.73	.53–1.0
	1–2					-.12	.16	.89	.65–1.2					-.11	.09	.90	.75–1.1
	3+					-.47	.26	.62	.37–1.0					-.56^**^	.19	.57	.40–.82
	Bullying																
	No	-.08	.07	.92	.80–1.1	-.39^*^	.15	.68	.50–.92					-.11	.09	.90	.75–1.1
	1 type	-.34^**^	.12	.71	.56–.90	-.47	.27	.62	.37–1.0					-.28	.17	.76	.54–1.1
	2 types	-.08	.15	.92	.69–1.2	.04	.33	1.0	.54–2.0					-.66^**^	.22	.52	.34–.79
	3 or 4 types	-.08	.16	.92	.67–1.3	-.26	.33	.77	.41–1.5					-.17	.25	.84	.51–1.4
	*Expressive*																
	Parent Status																
	Couple					-.33^**^	.12	.72	.56–.92	-.25^***^	.07	.78	.68–.90	-.28^***^	.06	.75	.67–.86
	Single					-.63^**^	.20	.53	.36–.79	-.15	.13	.86	.67–1.1	-.06	.12	.95	.74–1.2
	Siblings																
	0					-.21	.24	.81	.51–1.3					-.29^*^	.14	.75	.57–.98
	1–2					-.39^**^	.12	.68	.53–.86					-.23^***^	.07	.79	.69–.90
	3+					-.90^**^	.34	.41	.21–.78					-.18	.17	.84	.60–1.2
	Parent-adolescent relationship																
	Good					-.46^***^	.11	.63	.51–.79	-.23^***^	.07	.79	.70–.90	-.21^***^	.06	.81	.72–.91
	Bad					-.07	.31	.93	.51–1.7	-.19	.23	.83	.53–.1.3	-.63^**^	.21	.53	.35–.80
	Bully Victim																
	No					-.76^***^	.16	.47	.34–.64	-.42^***^	.09	.66	.55–.73	-.18^*^	.07	.84	.73–.96
	1 type					-.16	.20	.86	.58–1.3	-.25^*^	.12	.78	.61–.97	-.41^***^	.12	.66	.53–.83
	2 types					-.15	.24	.86	.53–1.4	.23	.15	1.3	.93–1.6	-.18	.16	.84	.63–1.1
	3 or 4 types					-.01	.26	.99	.60–1.6	.10	.19	1.1	.77–1.6	-.29	.19	.75	.51–1.1
	*Religious*																
	Parent Status																
	Couple	-.18^**^	.07	.83	.72–.96	-.28	.15	.76	.57–1.0	-.19^*^	.09	.83	.69–.99	-.18^*^	.08	.83	.71–.97
	Single	-.27^*^	.12	.76	.61–.96	-.19	.22	.82	.53–1.3	-.17	.16	.85	.61–1.2	-.02	.17	.98	.70–1.4
	Siblings																
	0					-.05	.28	.95	.55–1.6					-.26	.19	.77	.52–1.1
	1–2					-.36^*^	.16	.70	.52–.95					-.12	.09	.89	.75–1.1
	3+					-.10	.28	.91	.52–1.6					-.21	.17	.81	.59–1.1
	Parent-adolescent relationship																
	good	-.22^*^	.07	.80	.69–.91	-.25	.13	.79	.61–1.0	-.16	.08	.86	.73–1.0	-.13	.07	.88	.76–1.0
	bad	-.08	.16	.93	.70–1.3	-.38	.37	.69	.33–1.4	-.60	.34	.55	.29–1.1	-.58^*^	.27	.56	.33–..95
	Bully victim																
	No	-.08	.08	.92	.79–1.1	-.19	.16	.83	.60–1.1	-.33^**^	.11	.72	.58–.89	-.20^*^	.09	.82	.68–.98
	1 type	-.52^***^	.13	.60	.46–.77	-.42	.29	.65	.37–1.2	-.04	.16	.96	.70–1.3	-.33^*^	.16	.72	.53–.98
	2 types	-.41^*^	.17	.66	.48–.92	-.61	.36	.54	.27–1.1	-.22	.19	.80	.55–1.2	.09	.20	1.1	.73–1.6
	3 or 4 types	-.03	.16	.97	.71–1.3	.01	.31	1.0	.55–1.8	.18	.23	1.2	.77–1.9	.27	.23	1.3	.84–2.0
	**Baseline Behaviour**																
Model 3	Substance Use	3.3^***^	.12	27.2	21.7–34.2												
	Interaction Authority					1.9^***^	.16	6.8	4.9–9.4								
	Delinquency									2.0^***^	.10	7.6	6.3–9.2				
	Absenteeism													1.6^***^	.08	5.1	4.4–6.0

Models are controlling for sex, ethnicity, religion, parental education, household income, housing, and social class; significance is shown in the table ^*^ p < .05 ^**^ p < .01 ^***^ p < .001

Our findings showed that likelihood of reporting internalizing behavioural problems was increased (OR ~ 1.3) among the adolescents participating in expressive and religious activities. Expressive and religious involvement, however, decreased the odds (.66 < OR < .86) of reporting externalizing behavioural problems. While involvement with sports activities was associated with a decreased likelihood of reporting psychological/psychiatric distress, substance use, and problems with authority, it was associated with an increased likelihood of reporting absence from school.

Certain family factors (parent-adolescent relationship, household away from one or both parents, parental involvement) increased the adolescents’ likelihood of experiencing externalizing and internalizing disruptive behaviours. Bully victims were approximately two to eight times more likely to report disruptive behavioural problems than those adolescents who were not the victim of bullying.

### Model 2

In this model it was tested if participation in extracurricular activity moderated the effect of family and school factors on disruptive behavioural problems. The results (Tables 2 and 3) revealed that involvement in sports moderated the predictive effect of bullying on psychological/psychiatric distress and substance use. Expressive involvement moderated the effect of bullying on adolescents’ delinquency, absence from school, and psychological distress if the adolescents experienced only one type of bullying. Similarly, if the adolescents only experienced one type of bullying, participation in religious activities decreased the impact of bullying on substance use and absenteeism from school.

Expressive/religious involvement decreased the impact of siblings on interaction with authorities and/or absence from school. Those adolescents without siblings were less likely to report interaction with authorities if they were involved in sports activities and those adolescents with three or more siblings were less likely to experience absenteeism from school if they were involved in sports activities. Most importantly, participation in sports, expressive and religious activities was associated with a decreased likelihood to report externalizing behavioural problems among adolescents from intact families who had a good relationship with their parents. On the other hand, expressive activities seemed to enhance the effect of parent-adolescent relationships on psychological and psychiatric distress.

### Model 3

Model 3 (Tables 2 and 3) showed that adolescents who reported disruptive behavioural problems in 2004/2005 were more likely to also report these problems in 2006/2007. This was especially true for adolescence who used alcohol, drugs, or cigarettes. Adolescents who used any substance in 2004 were inclined (OR = 27.2) to use a substance in 2007 compared to their sober counterparts of 2004.

## Discussion

This study of English school-going adolescents shows that adolescent disruptive behavioural problems are most strongly predicted by prior acts of disruptive behaviour [5]. The findings highlight the effect social environmental factors have on adolescent problem behaviour by supporting findings from prior studies that the presence and strength of the relationship between extracurricular activity and developmental outcome of the youth depends on the activity and the outcome being studied. It corroborated findings from prior studies that participation in sports activities was associated with reduced likelihood of the presence of depression and anxiety symptoms in adolescents [56, 57], while young people who participated in expressive and religious activities reported higher levels of psychological symptoms [8]. Furthermore, similar to the findings of Bandura et al. [19] our findings included reduced odds of reporting disruptive behaviour for adolescents who were involved in religious/expressive activities. Deviating from the American literature [58], but comparative to international studies [24], adolescents’ volunteering activities did not affect the likelihood of reporting disruptive behavioural problems. This discrepancy could be due to a difference in conceptualization of volunteering or a difference in societal volunteering role/framework between USA and England.

The findings further show that adolescent’s family setting affects the likelihood of presenting with disruptive behavioural problems [34, 35, 59]. For instance, this study contributed to the extant body of literature showing that adolescents living harmoniously with both parents were less likely to report disruptive behavioural problems [60, 61]. Social interchanges in the school setting (bullying) were stronger predictors of disruptive behaviour than social interchanges in the family or extracurricular activity setting. This suggests a shift in social focus of the adolescent from family to school social environment. The school social environment took on a more prominent role in shaping the adolescents behaviour.

This study corroborates the results from earlier studies that negative social interactions can have serious consequences [62–66]. Results from the British National Survey of Psychiatric Morbidity [63] and the Avon Longitudinal Study of Parents and Children [66] show that bully victimization can have long-term effects. Given the health and mortality risk posed by disruptive behaviour early intervention in addressing these problems is important.

The results of this study support Bronfenbrenner’s developmental ecological model [43]: An interaction was found between the social interchanges of the extracurricular activity setting and the family/school setting. In some situations, youth involvement in extracurricular activities provided some protective benefits for disruptive behavioural problems. For instance, involvement in sports was able to buffer the effect of negative school social interactions on internalizing and externalizing behaviour. The findings of this study suggest that involvement in extracurricular activities would provide adolescents with modest protective behavioural skills if they were exposed to low levels of bullying. The impact of family and school stressors on display of disruptive behavioural problems was reduced by about 20 % if adolescent engaged in protective extracurricular activities.

In other situations, the involvement in extracurricular activities exacerbated disruptive behavioural problems. Results from this study show that participation in expressive and religious activities enhanced the impact of negative family and school social interactions on internalizing behaviour. The moderation effect could be explained by Bronfenbrenner & Morris’s argument [67] that adolescents will apply the social knowledge and skills acquired within one social setting to the other social settings they are involved in. Acquired social knowledge and skills will differ depending on the social setting attended. In addition, certain social skills might be beneficial in one setting, but could be counterproductive in other settings. This might explain the apparent contrast that involvement in some extracurricular activities is protective for adolescent behavioural problems, while involvement in other extracurricular activities encourages adolescents’ behavioural problems.

### Strengths and limitations

The LSYPE is a unique study following a representative sample of English adolescents over time. While a wealth of measures has been included in this longitudinal dataset, a limitation of the study is that only a few of these measures are consistently available over time. For instance, longitudinal measurement of peer and sibling social interactions is missing. Measures of peer influence would have enhanced the overall coverage of the adolescent’s social environment and could have confirmed the adolescent’s shift from family to peer environment. Information regarding family mental health problems would also have strengthened this study.

The focus of this study was on investigating the moderating effect of several different extracurricular activities. As intensity of participation was not measured in enough detail by the LSYPE, this study was unable to investigate how the moderating effect changes with increased participation (more frequent participation and/or participation in multiple extracurricular activities). Potential selection effects, such as intensively active youth experiencing different home and school settings thereby affecting disruptive behavioural problems differently, could not be substantiated. It can, however, be stated that the correlation between number of extracurricular activities an adolescent participated in and various family and school factors was weak (Goodman and Kruskal gamma .04–.15) or non-existent suggesting selection is not present.

Despite these limitations, the findings of this study reveal that participation in certain extracurricular activities is associated with a decreased adolescent engagement in specific disruptive behaviour.

## Conclusion

The results presented in this paper support Erikson’s and Bronfenbrenner’s reasoning; social interactions within the family, school and community setting shaped adolescents’ behaviour. This result merits further exploration into the potential intervention aspect of extracurricular activities. A future study could include extracurricular activities as one of its intervention strategies to tackle disruptive behavioural problems among adolescents. The findings could also shed some light on whether adolescents with certain developmental paths have a preference to participate in specific extracurricular activities. However, since the strongest predictor of disruptive behavioural problems at age 16 was display of disruptive behavioural problems at age 13, prevention campaigns in childhood among risk groups might prove to be more effective in reducing the adolescent prevalence of disruptive behavioural problems.
